# Supplementary material for: Yield and combining ability of temperate ex-PVP introgressed and tropical maize under contrasting moisture regimes
Source: PLoS One. 2026 Mar 6;21(3):e0344085. doi: 10.1371/journal.pone.0344085 (PMC12965617; doi:10.1371/journal.pone.0344085)
Supplement: Attachment — Submitted filename: REVIEWER COMMENTS ALERI ET AL MANUSCRIPT.pdf [file pone.0344085.s004.pdf]

**REVIEWER'S COMMENTS**

**Combining Ability and Yield Performance of Temperate ex-PVP Introgressed and Tropical Maize Lines under Contrasting Moisture Regimes**

1. Title is too long, if possible, shorten it
2. Short title is not very precise/clear
3. Key words: include the word maize lines if possible
4. Abstract: Sentences 6 and 7, on sites, evaluations were conducted in 8 sites/locations, with 6 WW and 2 DS. This improves readability.
5. TI in the abstract...to be written in full and then acronym in brackets, for later reference
6. The study was commenced in 2008, it's a long time now, so justify why it took so long to complete or publish this nice piece of research work. It reduces its novelty or validity somehow.
7. Line 126: Tropical not tropicalized
8. Line 137 -138: rephrase, not six sites in Kenya. First indicate total sites 8, then explain 6 WW and 2 DS. This is related to Point number 4 in abstract
9. Line 186: AGD-R, write in full
10. Line 212: Is it unit or unity, check???
11. Table 1: For L14 to L20, pedigree, description and attributes are congested and not aligning at some point. Check and correct. Or reduce content
12. Line 229-230 on GY, is it the average or across sites? Correct and make it clear for WW and DS yield
13. Line 267: Performance of testcross hybrids; somehow show the level of significance in the text (P value), not always but to somehow show readers the differences where significant what P value, before even check in the table of results.
14. Line 298...same as Point 13
15. Line 321 remove the double comma
16. Line 352, check x-PVP should be ex-PVP, also check and correct throughout the document
17. Line 391: make it clear that TI expands genetic base and improve yield in tropical germplasm
18. Line 404: remove double spacing before ....than
19. Line 437 and 448, Have a stand-alone Conclusions or concluding remarks. Separate Discussion and Conclusions
20. Line 437 and 448, repeated in conclusion, bring about monotony
21. Figures 1, 2 and 4 are not clear. Check if there is a way of making these clearer and readable
22. References: Some journal names are written in short, some in full. Be consistent.
